# Supplementary material for: Molecular structure of promoter-bound yeast TFIID
Source: Nat Commun. 2018 Nov 7;9:4666. doi: 10.1038/s41467-018-07096-y (PMC6220335; doi:10.1038/s41467-018-07096-y)
Supplement: Supplementary file 1 — Supplementary_Information-NatureComm-final-proof [file 41467_2018_7096_MOESM1_ESM.pdf]

Supplementary Information for

Molecular structure of promoter-bound yeast TFIID

Olga Kolesnikova, Adam Ben Shem, Jie Luo, Jeff Ranish, Patrick Schultz and Gabor Papai

Correspondence to: patrick.schultz@igbmc.fr and gabor.papai@igbmc.fr

Contents:

Supplementary Figures 1 to 10
Supplementary Table 1

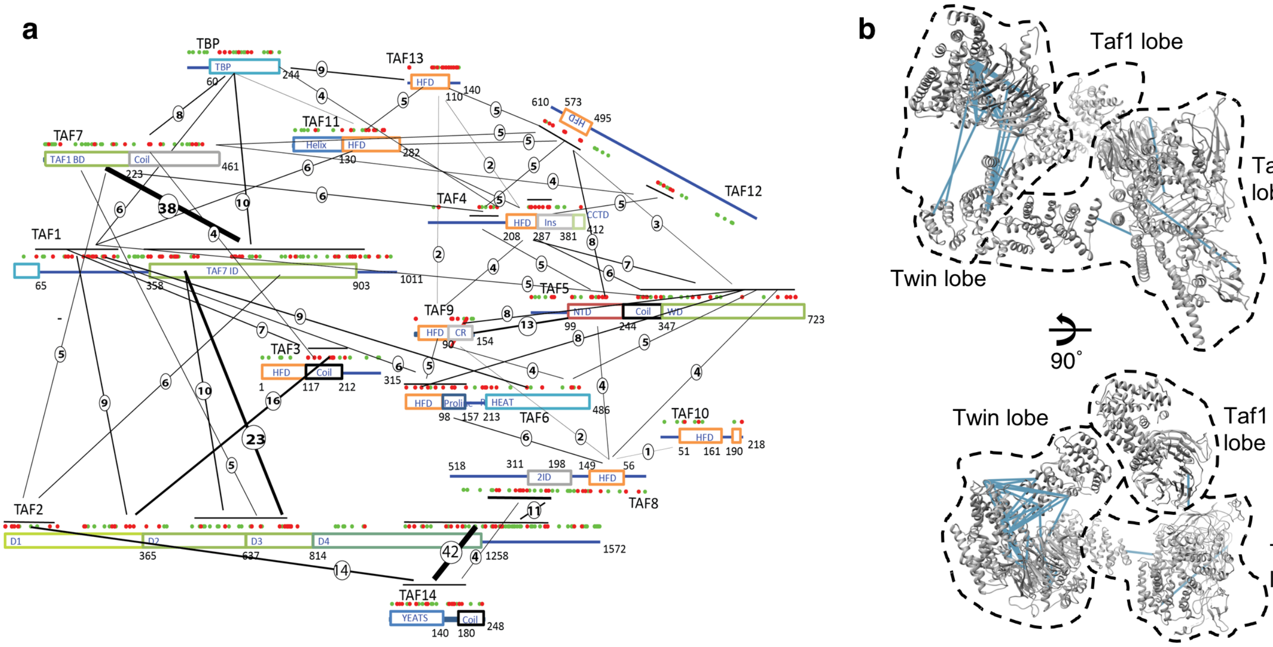

Supplementary Figure 1: Region specific cross-linking map

a, Intersubunit TFIID cross-linking map. Conserved protein domains are highlighted by colored boxes, black lines above the sequences indicate a group of residues that display a similar cross-linking pattern. Red dots indicate cross-linked lysine residues; green dots indicate unmodified lysine residues. Black connections and the circled number associated to each connector represent crosslinks and the number of different crosslinks between associated domains or subunits. **b**, Crosslinked residues mapped on the structure. The majority of crosslinked residues reside in loops or poorly ordered regions that could not be mapped precisely within the structure. Only those links with both residues in the modelled regions are shown.

a [6FAM]TGTCATGAGATTATTGGAAACCACCAGAATCGAATATAAAAGGC-
GAACACCTTTCCCAATTTTGGTTTCTCCTGACCCAAAGACTTTAAATTTAATTTA

b

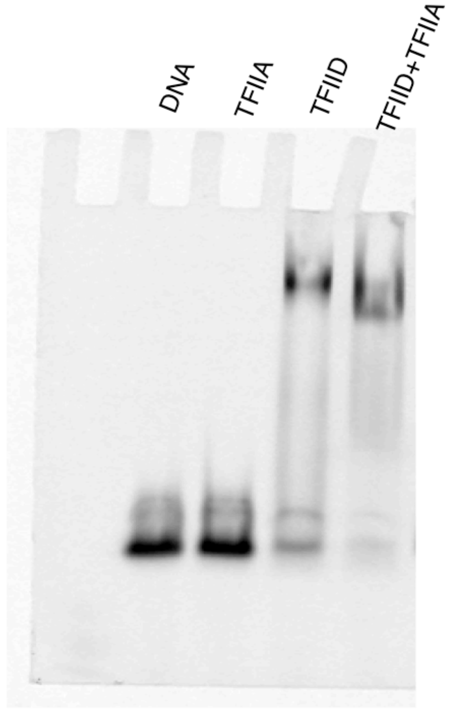

Supplementary Figure 2: Binding of the TFIID complex to pGAP

a, Sequence of the 6-Carboxyfluorescein (6-FAM*)* labelled glyceraldehyde-3-phosphate dehydrogenase promoter (pGAP) used for the TFIID binding studies. **b,** Fluorescent analysis of 6-FAM labeled pGAP migration on agarose-polyacrylamide gels in the presence of TFIIA and TFIID.

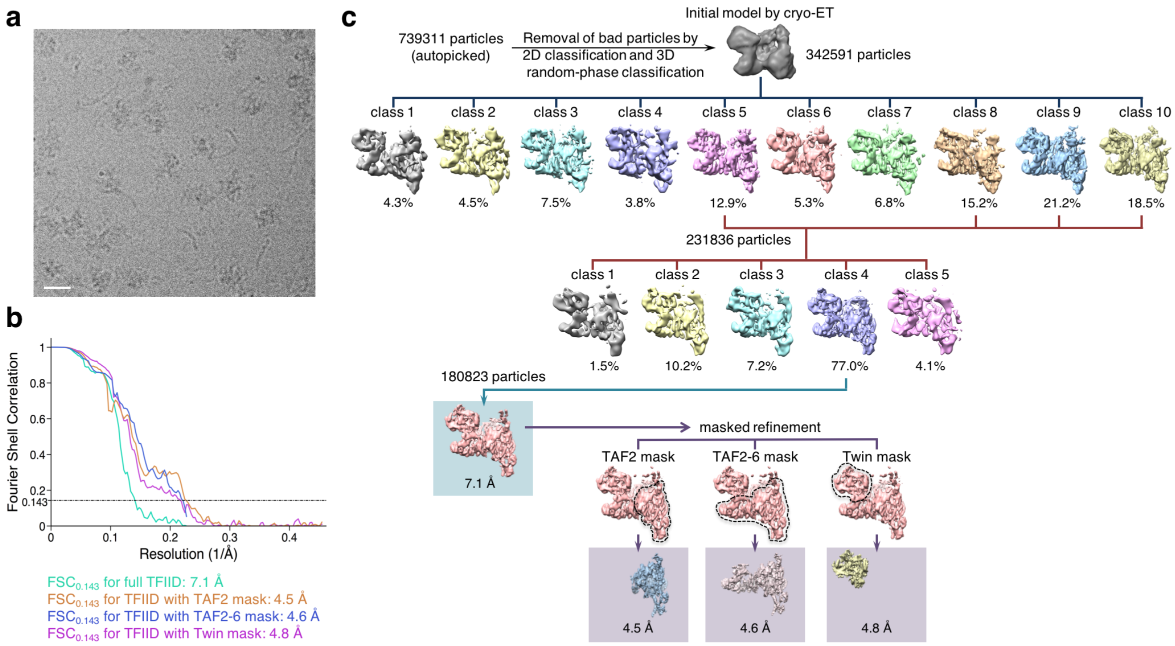

Supplementary Figure 3: Single-particle cryo-EM analysis of the TFIID/TFIIA/pGAP complex

a, Original images of frozen-hydrated Kp TFIID/TFIIA/pGAP complexes. Scale bar represents 20 nm. **b,** Fourier Shell Correlation curves as a function of spatial frequencies in 1/Å for the entire TFIID complex (cyan), the Taf2-lobe (orange), the Taf2-lobe including the Taf6 HEAT repeats (blue) and the Twin-lobe (pink). **c,** Three-dimensional classification scheme for the TFIID-TFIIA-pGAP complex.

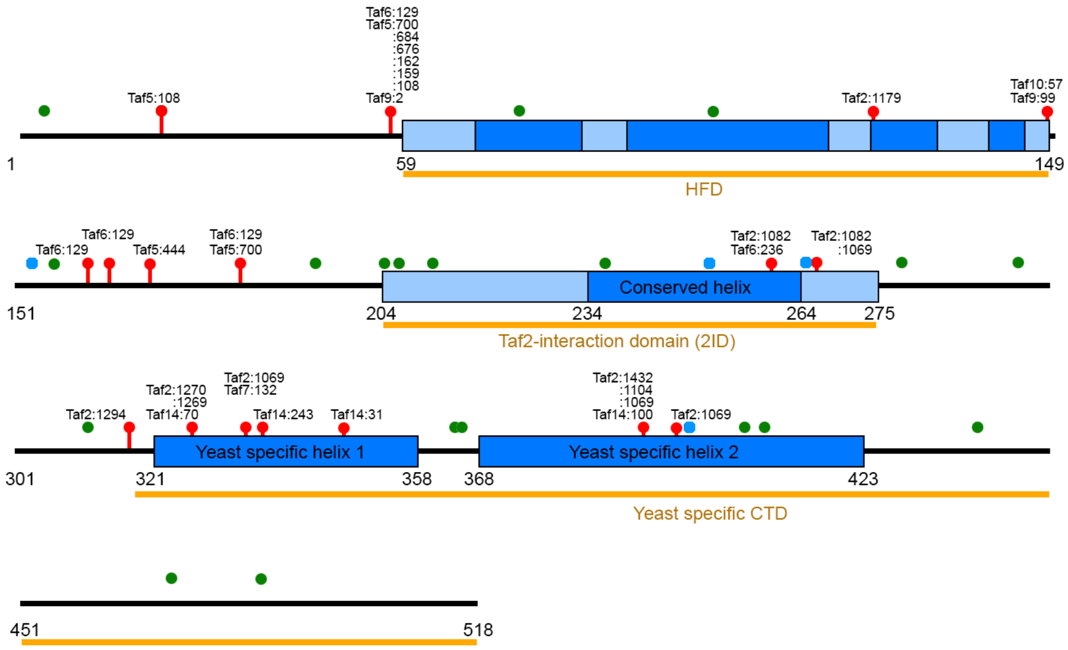

Supplementary Figure 4: Sequence alignment, secondary structure prediction and cross-linking of Kp Taf8

Schematic sequence alignment showing that the histone fold domain (HFD) and the Taf2-interaction domain (2ID) are conserved through evolution while yeast species contain a specific C-terminal domain. Predicted helices are shown in dark blue. Cross-linked subunits are indicated, inter-subunit cross-links are shown by red dots, additional intra-subunit crosslinks are in blue, and non-cross-linked lysines are highlighted in green.

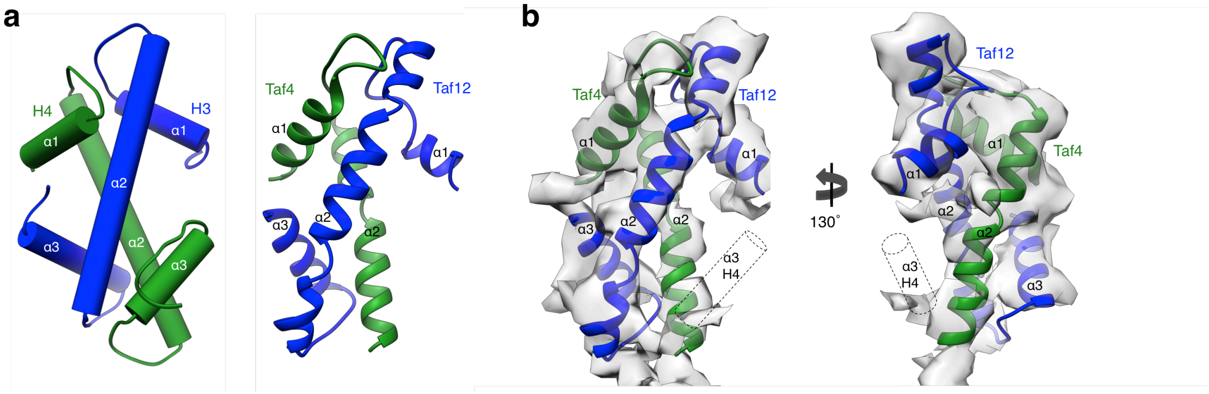

Supplementary Figure 5: Structure of the Taf4-Taf12 heterodimer

a, Comparison of the bona-fide H3-H4 histone fold with the crystal structure of the human TAF4-TAF12 histone-like heterodimer showing the missing α3 helix in TAF4. **b,** Fitting of the TAF4-TAF12 structure into the cryo-EM density showing that the α3 helix of Taf4 is also missing in holo TFIID.

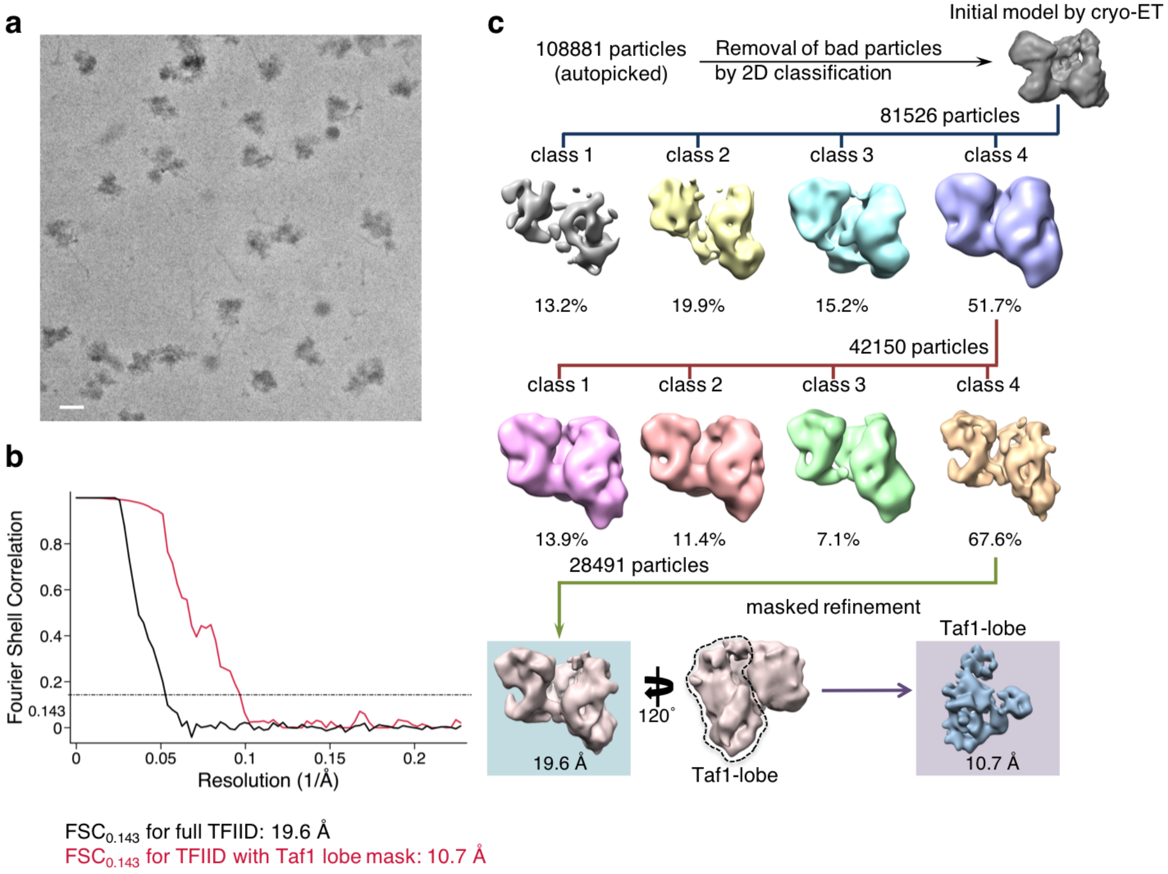

Supplementary Figure 6: Volta Phase Plate cryoEM imaging

a, Original images of frozen-hydrated Kp TFIID/TFIIA/pGAP complexes recorded with Volta Phase Plate. Scale bar represents 20 nm. **b,** Fourier Shell Correlation curves as a function of spatial frequencies in 1/Å for the entire TFIID complex (black) and the Taf1-lobe (red). **c,** Three-dimensional classification scheme for the TFIID-TFIIA-pGAP complex.

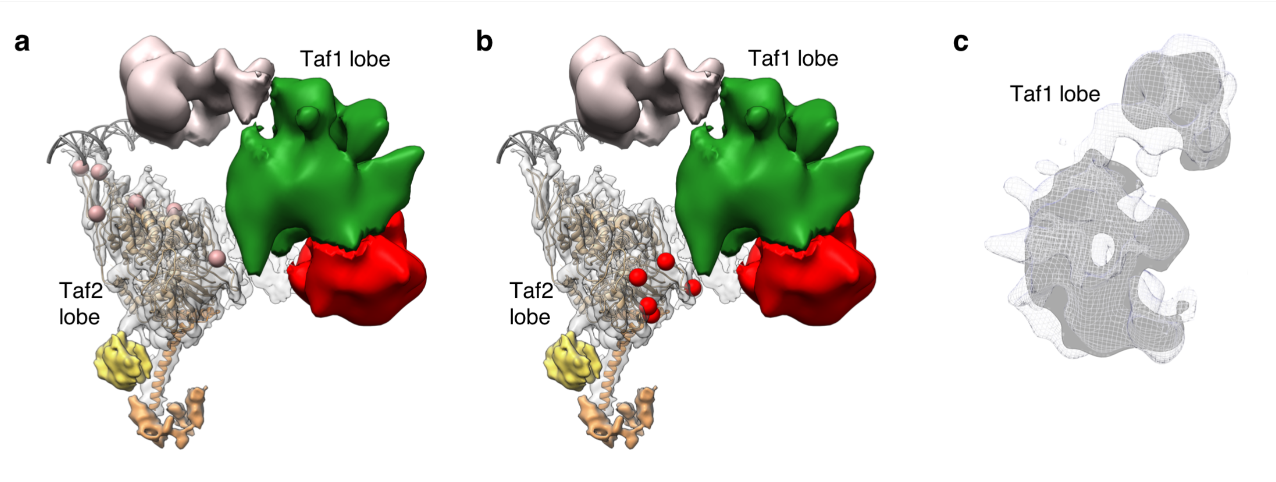

Supplementary Figure 7: Positioning subunits in Taf1-lobe

a, Taf2 lysine residues that cross-linked to Taf1 are highlighted as pink balls on the docked atomic structure of Taf2; **b,** Taf2 lysine residues that cross-linked to Taf3 are highlighted as red balls on the docked atomic structure of Taf2. The Taf1-lobe is represented in solid pink-green-red. **c,** ring-like density shown by the cross section (solid grey) of Taf1-lobe (in mesh).

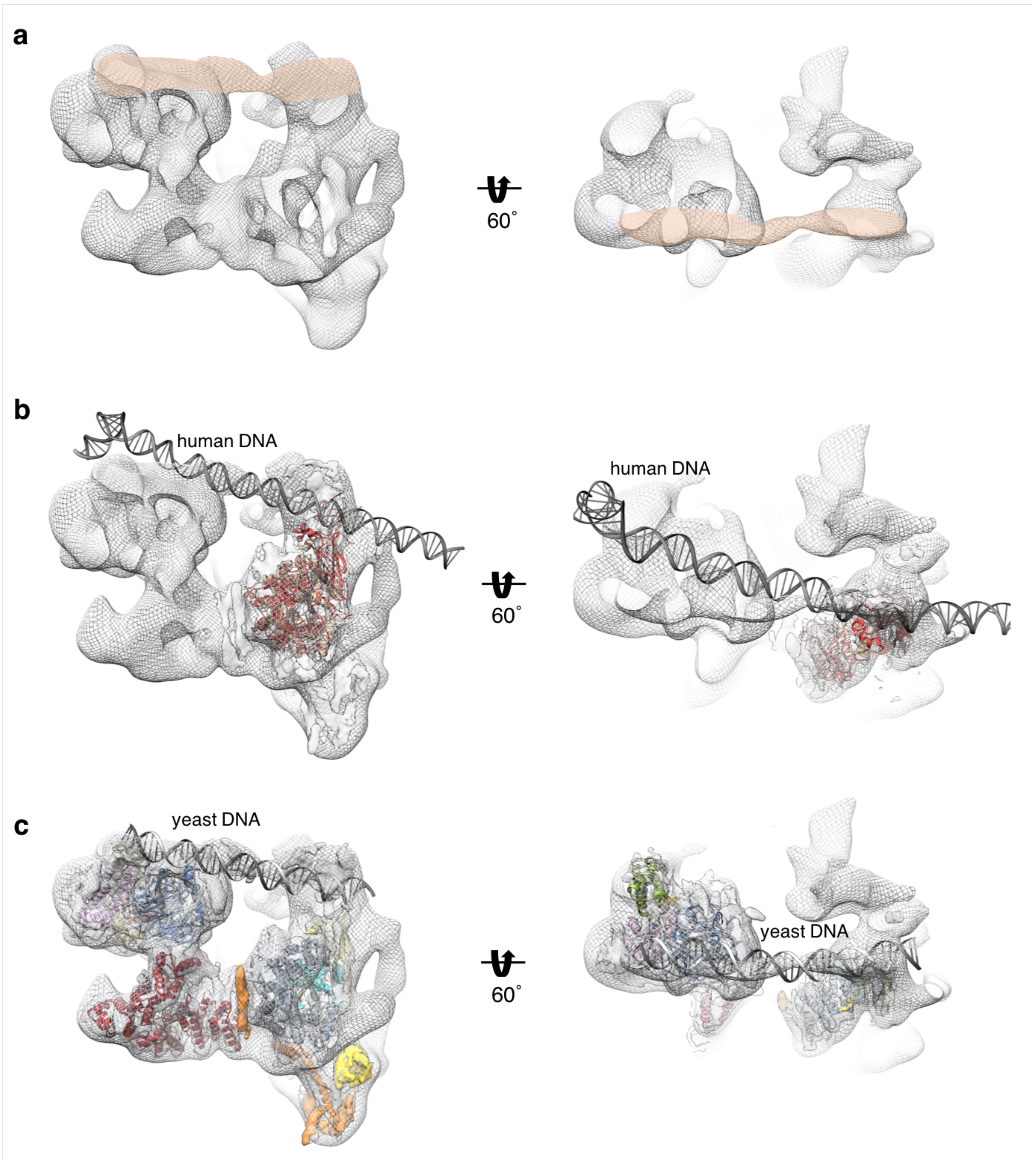

Supplementary Figure 8: Interaction of yeast and human TFIID with DNA

a, The EM density of *Kp*TFIID was low-pass filtered to 20 Å to visualize the path of the DNA (in orange); **b,** Taf2 from the human TFIID structure (pdb: 5fur in red) is superimposed its yeast counterpart (in tan). The human synthetic promoter DNA fragment in 5fur is depicted in dark grey ladder. The low-pass filtered EM density of *Kp*TFIID (in mesh) was aligned with the 4.5 Å resolution Taf2-lobe structure (transparent white). Densities are present along the path of the human DNA in the yeast TFIID. **c,** Modelled *Kp*TFIID subunits with DNA (depicted in grey-white ladder) in the 20 Å filtered EM structure. DNA follows the densities found along the path of human DNA.

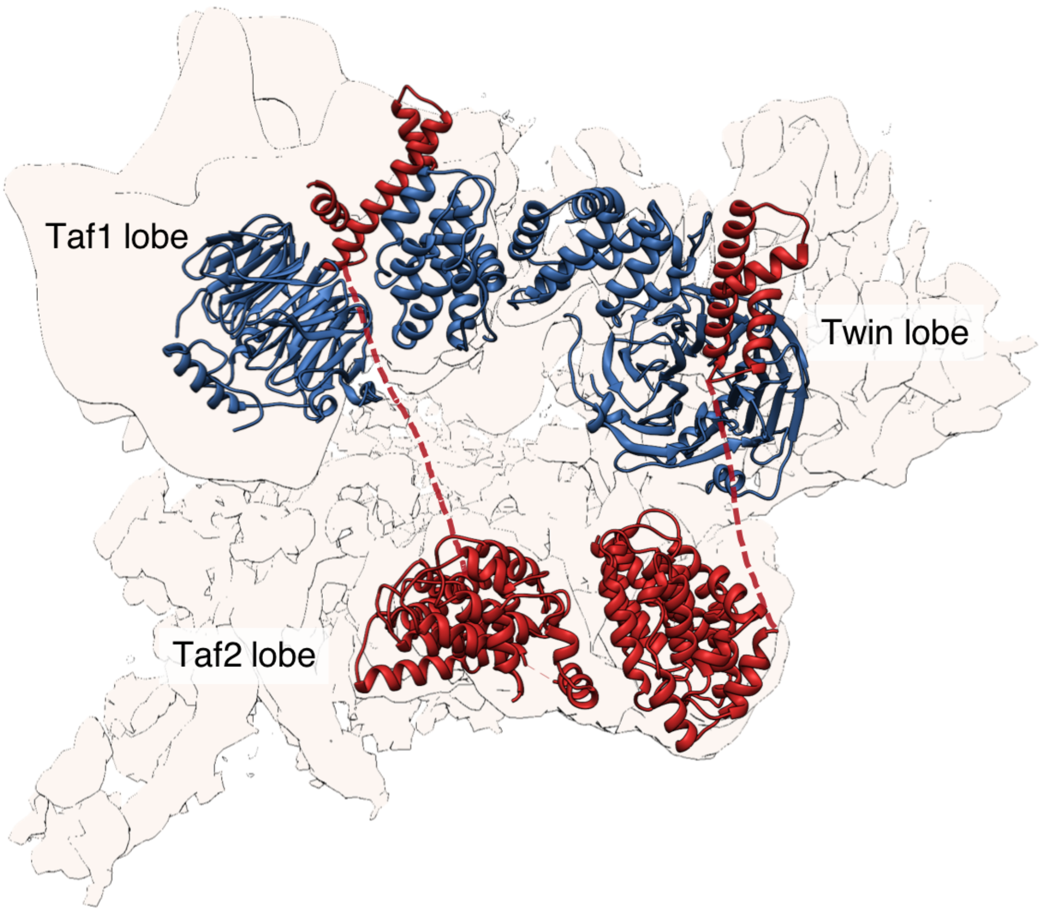

Supplementary Figure 9: Circular arrangement of the Taf5 and Taf6 TFIID subunits

Taf5-Taf6 heterotetrameric arrangement forming a topologically closed structure that connects the three TFIID lobes. Taf5 is represented in blue and Taf6 in red.

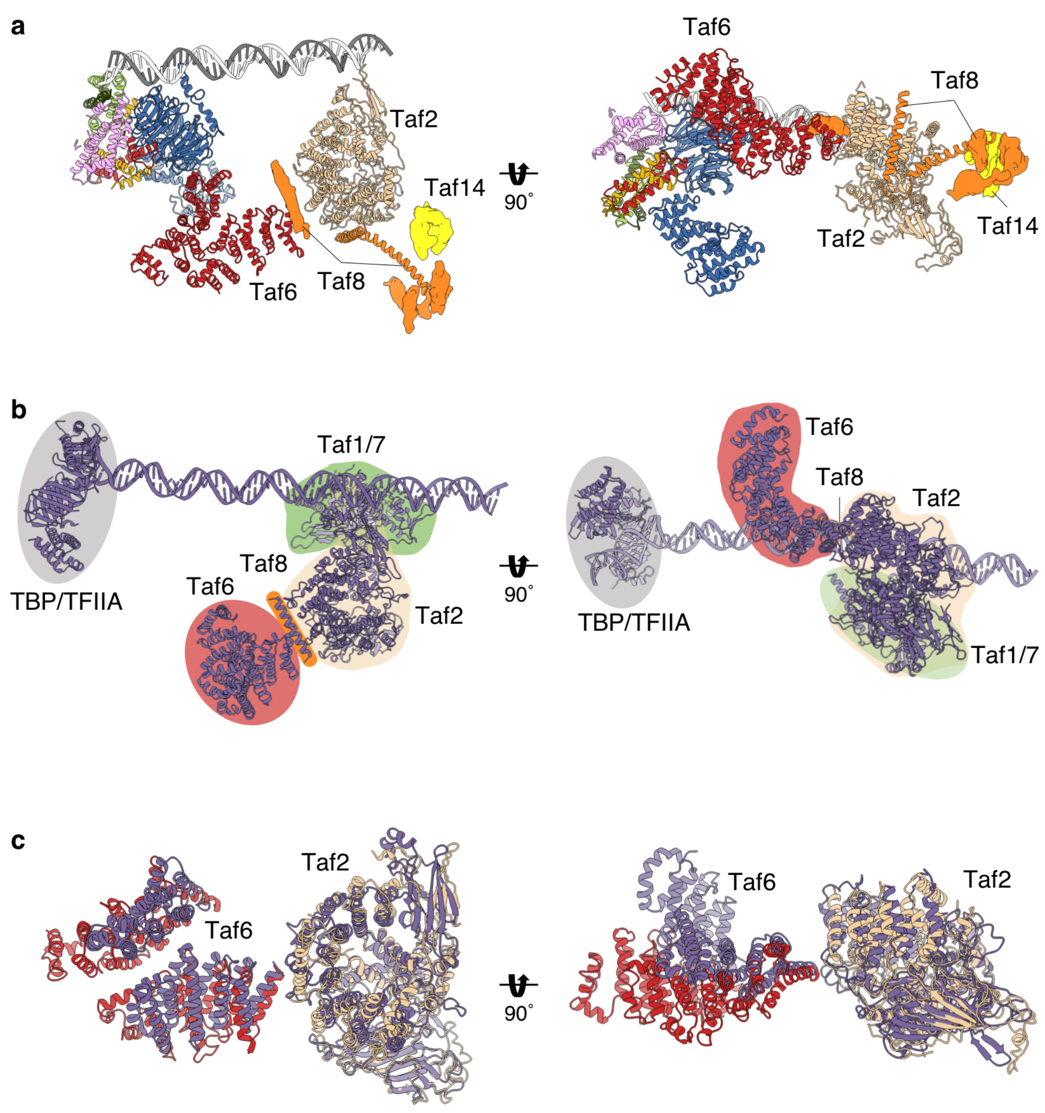

Supplementary Figure 10: Comparison of human and yeast TFIID

a, Modelled *Kp*TFIID subunits in the EM density; **b,** modelled human TFIID subunits (pdb: 5fur); **c,** Common modelled yeast and human TFIID subunits (Taf2 and Taf6) aligned. The Taf6 dimerization interface is changed in yeast compared to the human TFIID.

Supplementary Table 1

Cryo-EM data collection, refinement and validation statistics

	KpTFIID EMDB-0249	KpTFIID-DNA-TFIIA EMDB-0251	Taf1 Lobe - Phase Plate EMDB-0250	Modelled coordinates (PDB 6HQA)
**Data collection and processing**				
Magnification	50000	105000	105000	
Voltage (kV)	300	300	300	
Electron exposure (e–/Å^2)	50			
Defocus range (μm)	0.8-4.2	0.8-4.9	0.4-0.7	
Pixel size (Å)	1.1	1.1	1.1	
Symmetry imposed	C1	C1	C1	
Initial particle images (no.)	255723	295734	162598	
Final particle images (no.)	155620	180823	28491	
Map resolution (Å)	12.13	7.1	10.7	
FSC threshold	0.143	0.143	0.143	
Map resolution range (Å)				
**Refinement**				
Model composition				
Non-hydrogen atoms				12700
Protein residues				2551
Ligands				
R.m.s. deviations				
Bond lengths (Å)				0.004
Bond angles (°)				0.924
Validation				
MolProbity score				2.44
Clashscore				14.65
Poor rotamers (%)				0
Ramachandran plot				
Favored (%)				77.16
Allowed (%)				15
Disallowed (%)				7.55
