# Supplementary material for: Molecular structure of promoter-bound yeast TFIID
Source: Nat Commun. 2018 Nov 7;9:4666. doi: 10.1038/s41467-018-07096-y (PMC6220335; doi:10.1038/s41467-018-07096-y)
Supplement: Supplementary file 3 — Description of Additional Supplementary Files [file 41467_2018_7096_MOESM3_ESM.pdf]

Description of Additional Supplementary Files

File Name: Supplementary Data 1
Description: Mass spectrometry analysis of the purified endogenous *Komagataella phaffii* (*Pichia pastoris*) TFIID.

File Name: Supplementary Data 2
Description: Inter- and intra-subunit crosslinks of TFIID defined by crosslinking-mass spectrometry.
